# First-Line Matched Related Donor Hematopoietic Stem Cell Transplantation Compared to Immunosuppressive Therapy in Acquired Severe Aplastic Anemia

**DOI:** 10.1371/journal.pone.0018572

**Published:** 2011-04-25

**Authors:** Frank Peinemann, Ulrich Grouven, Nicolaus Kröger, Carmen Bartel, Max H. Pittler, Stefan Lange

**Affiliations:** 1 Institute for Quality and Efficiency in Health Care (IQWiG), Cologne, Germany; 2 Hannover Medical School, Hannover, Germany; 3 Interdisciplinary Clinic for Stem Cell Transplantation, University Hospital Hamburg-Eppendorf, Hamburg, Germany; 4 German Cochrane Centre, University of Freiburg, Freiburg, Germany; Centre de Recherche Public de la Santé (CRP-Santé), Luxembourg

## Abstract

**Introduction:**

Acquired severe aplastic anemia (SAA) is a rare and progressive disease characterized by an immune-mediated functional impairment of hematopoietic stem cells. Transplantation of these cells is a first-line treatment option if HLA-matched related donors are available. First-line immunosuppressive therapy may be offered as alternative. The aim was to compare the outcome of these patients in controlled trials.

**Methods:**

A systematic search was performed in the bibliographic databases MEDLINE, EMBASE, and The Cochrane Library. To show an overview of various outcomes by treatment group we conducted a meta-analysis on overall survival. We evaluated whether studies reported statistically significant factors for improved survival.

**Results:**

26 non-randomized controlled trials (7,955 patients enrolled from 1970 to 2001) were identified. We did not identify any RCTs. Risk of bias was high except in 4 studies. Young age and recent year of treatment were identified as factors for improved survival in the HSCT group. Advanced age, SAA without very severe aplastic anemia, and combination of anti-lymphocyte globulin with cyclosporine A were factors for improved survival in the IST group. In 19 studies (4,855 patients), summary statistics were sufficient to be included in meta-analysis. Considerable heterogeneity did not justify a pooled estimate. Adverse events were inconsistently reported and varied significantly across studies.

**Conclusions:**

Young age and recent year of treatment were identified as factors for improved survival in the transplant group. Advanced age, SAA without very severe aplastic anemia, and combination of anti-lymphocyte globulin with cyclosporine A were factors for improved survival in the immunosuppressive group. Considerable heterogeneity of non-randomized controlled studies did not justify a pooled estimate. Adverse events were inconsistently reported and varied significantly across studies.

## Introduction

Acquired severe aplastic anemia (SAA) is a rare [1] and potentially fatal disease which is characterized by hypocellular bone marrow and pancytopenia, and mainly affects young adults. The incidence rate was estimated at less than 4 per million people per year [2]. The major signs and symptoms are severe infections, bleeding, and exhaustion. The underlying pathophysiology is thought to be an aberrant immune response involving the T-cell mediated destruction of hematopoietic stem cells. In most cases, the cause is unknown, although various triggers such as drugs, toxins, and viruses have been reported [3], [4].

The treatment of SAA mainly includes immunosuppressive therapy (IST) with antithymocyte globulin (ATG)/antilymphocyte globulin (ALG) and cyclosporine A (CSA), or allogeneic hematopoietic stem cell transplantation (HSCT) [3], [4], [5]. Allogeneic HSCT is seen as the treatment of choice for selected patients with an HLA-matched related donor [6], [7]. Allogeneic HSCT is associated with graft failure, graft-versus-host disease (GVHD), and organ toxicities. On the other hand, patients may not respond to IST and long-term IST is associated with the development of clonal diseases [8]. Clinical treatment algorithms have been suggested to find a decision that meets individual conditions, personal preferences, and prognostic factors [9].

The present systematic review and meta-analysis compares the outcome after first-line HLA-matched related donor HSCT vs. IST in SAA patients in published controlled trials.

## Methods

While preparing this systematic review and meta-analysis, we endorsed the PRISMA statement, adhered to its principles and conformed to its checklist [10], [11].

### Study inclusion criteria

We included patients with acquired severe aplastic anemia who received, as first-line treatment, allogeneic HSCT from HLA-matched related donors (MRD) as the test intervention and IST as the control intervention. Study design was limited to randomized controlled trials (RCTs) and non-randomized intervention studies [12]. We did not set a minimum sample size to be considered. Full-text publications in English language were considered. We set no limits on year of publication or year of treatment. A protocol is not available.

### Search strategy

MEDLINE (1950 to 2010), EMBASE (1980 to 2010) and The Cochrane Library (to 2010) were searched without restrictions on study design and publication year (final search 10 January 2010). The first database search was conducted 24 January 2006. The final search 10 January 2010 included a modified strategy to consider MeSH changes and to render the exclusion of animal studies more precisely. The MeSH term BONE MARROW TRANSPLANTATION was deleted from one category [13]. For MeSH 2008 there was a major revision of Publication Types (PT) and the phrase “as Topic” was added [14]. We introduced (ANIMALS not (ANIMALS and HUMANS)).sh. and replaced (ANIMALS not HUMANS).sh. To account for these changes, both searches were not restricted to any publication year. The terms and the syntax used for the search in MEDLINE via Ovid as shown in Table 1 were tailored to the requirements of the other 2 databases. Reference lists of all included original articles and 5 recent reviews (2007 to 2009) [8], [9], [15], [16], [17] were hand-searched. Abstracts of the American Society of Hematology Annual Meeting 2004 to 2009 [18] and information on studies registered at ClinicalTrials.gov [19] were searched online (April 2010).

**Table 1:** Search strategy used in MEDLINE via Ovid.

Database: Ovid MEDLINE(R) In-Process & Other Non-Indexed Citations, Ovid MEDLINE(R) Daily and Ovid MEDLINE(R) <1950 to Present>
Search Strategy: row number, search term (number of retrieved records)
1 exp ANEMIA, APLASTIC/(12718)
2 (aplast$ anem$ or aplast$ anaem$).tw,kf,ot. (6906)
3 or/1–2 (14343)
4 exp STEM CELL TRANSPLANTATION/(33962)
5 exp BONE MARROW TRANSPLANTATION/(37050)
6 exp TRANSPLANTATION, HOMOLOGOUS/(68947)
7 transplant$.tw,kf,ot. (271774)
8 graft$.tw,kf,ot. (187569)
9 (allograft$ or allo-graft$).tw,kf,ot. (42157)
10 (homograft$ or homo-graft$).tw,kf,ot. (4829)
11 or/4–10 (443058)
12 RANDOMIZED CONTROLLED TRIALS AS TOPIC.sh. (63488)
13 RANDOMIZED CONTROLLED TRIAL.pt. (279602)
14 random$.tw,kf,ot. (484356)
15 CONTROLLED CLINICAL TRIAL.pt. (79896)
16 RANDOM ALLOCATION.sh. (66150)
17 DOUBLE BLIND METHOD.sh. (102937)
18 SINGLE BLIND METHOD.sh. (13347)
19 (ANIMALS not HUMANS).sh. (3331490)
20 exp CLINICAL TRIALS AS TOPIC/(221553)
21 CLINICAL TRIAL.pt. (452572)
22 (clin$ adj25 trial$).tw,kf,ot. (173070)
23 ((singl$ or doubl$ or trebl$ or tripl$) adj25 (blind$ or mask$)).tw,kf,ot. (105483)
24 PLACEBOS.sh. (28204)
25 placebo$.tw,kf,ot. (122048)
26 RESEARCH DESIGN.sh. (57641)
27 COMPARATIVE STUDY.pt. (1453016)
28 exp EVALUATION STUDIES AS TOPIC/(779364)
29 FOLLOW-UP STUDIES.sh. (391452)
30 PROSPECTIVE STUDIES.sh. (267034)
31 (control$ or prospectiv$ or volunteer$).tw,kf,ot. (2222508)
32 (metaanaly$ or (meta and analy$) or ((review or search$) and (medical database$ or medline or pubmed or embase or cochrane or systemat$))).tw,kf,ot. (80827)
33 META-ANALYSIS AS TOPIC.sh. (9714)
34 META-ANALYSIS.pt. (22757)
35 exp REGISTRIES/(37774)
36 (registr$ or register$ or ibmtr$ or ebmt$).tw,kf,ot. (129832)
37 ((group or regist$) and (blood or stem cell or marrow) and transplant$ and (europ$ or international)).tw,kf,ot. (1120)
38 or/12–37 (7025740)
39 (ANIMALS not (ANIMALS and HUMANS)).sh. (3331490)
40 and/3,11,38 (1160)
41 40 not 39 (1087)
42 from 41 keep 1–1087 (1087)

### Study selection

**Figure 1:**
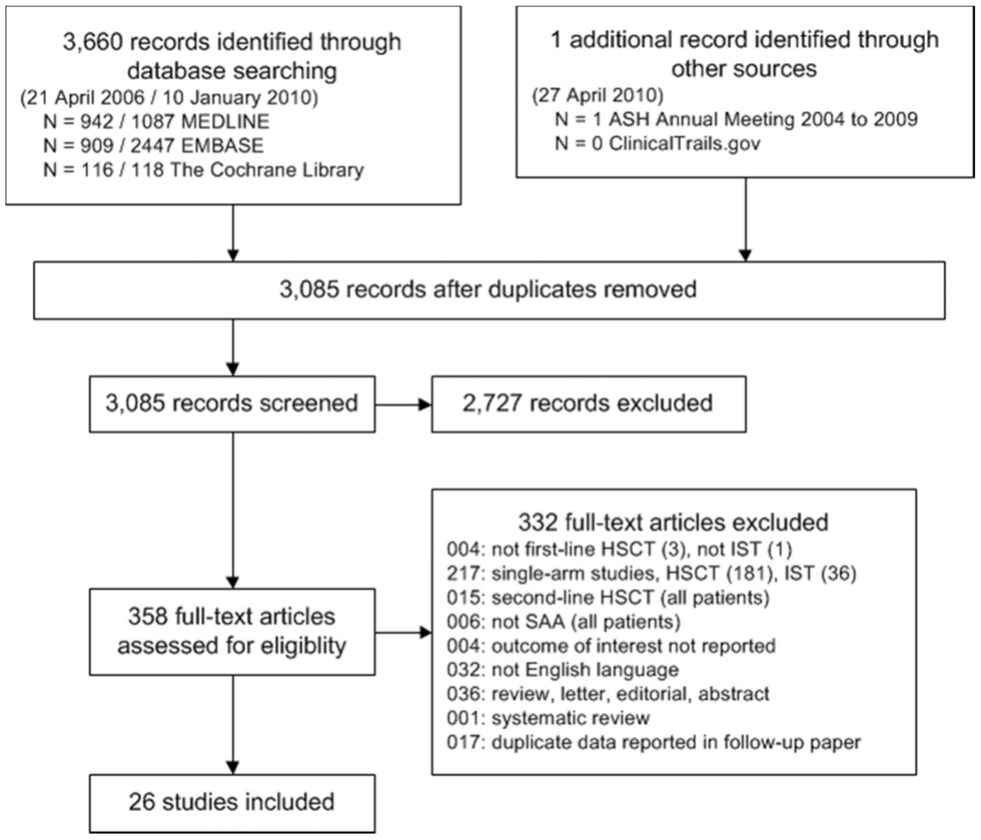
Literature search and study flow. Abbreviations: HSCT: hematopoietic stem cell transplantation; IST: immunosuppressive therapy; SAA: severe aplastic anemia.

First, articles were excluded if the title and/or the abstract clearly referred to other diagnoses than severe aplastic anemia and in addition clearly referred to other interventions than allogeneic hematopoietic stem cell transplantation. Second, articles not excluded in the first step were evaluated whether patients were analyzed in a test group after first-line allogeneic HSCT from a MRD and were compared with patients after first-line IST in a control group. Reporting of extractable information about overall survival was required for all included studies. For each excluded study, an appropriate reason was documented (Figure 1). All steps of the literature screening process were performed by two independent reviewers. Any disagreements were resolved by discussion. Criteria for classification of severity of aplastic anemia and quality of response after immunosuppressive treatment were applied according to the EBMT [21] [20].

### Risk of bias

Risk of bias within studies was evaluated by assessing study design, such as retro- or prospective planning, concurrent control group, criteria for assignment of patients to treatment arms, control for confounding factors, and other criteria, such as unclear selection of patients and analysis of the same patients in both treatment groups, that may increase the risk of bias especially in non-randomized trials [21]. A low risk of bias required a ‘yes’ for all three of the following topics: concurrent control group, control for confounding factors, and no other risk of bias factors.

Risk of bias across studies was evaluated by assessing publication bias and outcome reporting bias. We conducted funnel plots using hazard ratios and related standard errors of each meta-analysis to assess publication bias. We evaluated potentially relevant studies to identify studies that may have been excluded because of missing or insufficient outcome reporting. We evaluated published study protocols to identify outcome reporting different from appropriate procotols.

### Primary outcome: overall survival

The primary effect measure for meta-analyses was the hazard ratio. If the hazard ratio was not directly given in the publication, we extracted summary statistics from Kaplan Meier survival functions and estimated hazard ratios according to methods proposed by Parmar 1998 [22]. For estimation, we applied a tool which uses *p*-values of the appropriate log-rank test comparing the two survival functions of interest, number of patients analyzed, and number of events on each arm [23]. If this information was not available, hazard ratios were deduced from the graphical display of the survival curves, if possible. Meta-analyses were conducted using the generic variance approach [24], [25] and the random effects model [26]. Calculations were conducted using SAS version 9.2 (SAS Institute Inc., Cary, North Carolina, USA). The results of the meta-analyses were graphically displayed by means of a forest plot. Heterogeneity of the results was visually assessed and quantified using the I^2^ value [27]. A funnel plot was prepared by using The Cochrane Collaboration's Review Manager 5 (http://www.cc-ims.net/revman). In the case of considerable heterogeneity (I^2^≥50%), a pooled estimate is not reasonable and, therefore, was not calculated [28].

### Subgroup analysis

Sensitivity analysis: We tried to explain heterogeneity identified in the meta-analysis of overall survival by evaluating dichotomized subpopulations of study and patients characteristics in several sensitivity analyses [29]. Subgroups in individual studies: We extracted overall survival data of subgroups to reproduce survival functions from some individual studies if reported for both treatments. Data on subgroup analyses were extracted if available. The difference between the estimates of 2 subgroups was evaluated by conducting a test of interaction based on meta-regression models [30]. Calculations were conducted using SAS version 9.2 (SAS Institute Inc., Cary, North Carolina, USA).

### Factors for improved survival

We extracted statistically significant results from multivariate analyses on factors for improved survival, such as age, disease severity, treatment period, and type of IST, if reported in the included studies.

### Data collection and analysis

All steps of the data collection process were performed by two independent reviewers. Any disagreements were resolved by discussion. We collected study characteristics such as the number and region of participating centers, the treatment period, the number of analyzed patients per treatment arm, the proportion of patients with diseases other then SAA, a regimen other than first-line treatment, and median follow-up. Median age, gender, and time interval from diagnosis to treatment were extracted as patients' characteristics. For the HSCT arm we extracted the number of patients who were treated with conditioning irradiation and conditioning ATG or ALG. For the IST arm we extracted the number of patients treated with ATG or ALG alone or in combination with CSA.

The primary outcome was overall survival from the beginning of HSCT or IST. Kaplan Meier estimates were extracted directly from the text or deduced from survival curves given in the publication. The principal summary measure was the hazard ratio as specified in the meta-analysis section. We extracted the *p*-value of the log-rank test of the overall survival functions and the 5-year estimate of both treatment arms. Results from subgroup analyses were also extracted.

The secondary outcomes were overall mortality, graft failure, GVHD, no response to IST, and health-related quality of life. Graft failure included both primary and secondary types; acute GVHD was considered if grade II–IV was reported and chronic GVHD was considered if an extensive course was reported.

## Results

### Search results

Of 3,085 retrieved publications, 358 full-text papers were obtained for further assessment and 26 non-randomized controlled clinical trials were identified that met the inclusion criteria and were included in the present study [31], [32], [33], [34], [35], [36], [37], [38], [39], [40], [41], [42], [43], [44], [45], [46], [47], [48], [49], [50], [51], [52], [53], [54], [55], [56]. We did not identify any RCTs.

An overview of the study characteristics is presented in Table 2. Patients were recruited between 1970 and 2002 in 24 studies, whereas in 2 studies, year of treatment was not reported. In 13 studies [31], [34], [35], [39], [41], [42], [43], [44], [45], [46], [47], [49], [54], disease severity (SAA or VSAA) or first-line treatment was clearly classified for all patients. The proportion of patients with moderate aplastic anemia, unknown disease severity, or second-line treatment was less than 20% in 6 studies [32], [36], [38], [40], [50], [52] and was 20% or more in 7 studies [33], [37], [48], [51], [53], [55], [56]. 25 included trials used information about the availability of MRD to allocate patients to comparison groups, called *Mendelian (genetic) randomization* [57], [58]. In 3 studies [41], [52], [53], relevant data were not reported for the total study population but were reported separately for subgroups.

**Table 2:** Study characteristics.

No	Included studies	Group, region (N. centers)	Treatment period in years	N. analyzed patients; subgroups indicated HSCT vs. IST	N. patients other* than SAA (%); HSCT vs. IST	N. patients other† than first-line treatment (%); HSCT (MRD) vs. IST	Median follow-up in years
1	Ahn 2003 [31]	Seoul, Korea (10)	1990–2001	64 vs. 156	0 vs. 0	0 vs. 0	
2	Arranz 1994 [32]	Madrid, Spain (1)	1983–1991	21 vs. 29. Subgroup age 20–40 ys: 11 vs. 13	0 vs. 2 (7)	5 (24) vs. 1 (3)	–
3	Bacigalupo 1988 [33]	EBMT, Europe (57)	1981–1986	218 vs. 291. Subgroup age + PMN: <20 ys + <0.2 G/L: 55 vs. 46; <20 ys + ≥0.2 G/L: 63 vs. 57; ≥20 ys + <0.2 G/L: 32 vs. 89; ≥20 ys + ≥0.2 G/L: 44 vs. 36	45 (21) vs. 92 (32)	47 (22) vs. 0	2.6 vs. 2.7
4	Bacigalupo 2000 [34]	EBMT, Europe (?)	1976–1998	Subgroup year (IST = ALG) 1976–1989: 915 vs. 523; 1990–1998: 844 vs. 116. Subgroup year (IST = ALG+CSA) 1976–1989: 915 vs. 56; 1990–1998: 844 vs. 53	0 vs. 0	0 vs. 0	–
5	Bayever 1984 [35]	Los Angeles, USA (1)	1977–1982	35 vs. 22	0 vs. 0	0 vs. 0	–
6	Champlin 1984 [36]	Los Angeles, USA (1)	unclear	61 vs. 69. Subgroup age <20: 35 vs. 21; 20–<35: 21 vs. 21; ≥35: 5 vs. 27	0 vs. 0	0 vs. 5 (7)	–
7	De Planque 1990 [37]	Leiden, Netherlands (1)	1974–1987	19 vs. 63	0 vs. 0	8 (42) vs. 19 (30)	2.8 vs. 1.8
8	Doney 1997 [38]	Seattle, USA (1)	1978–1991	168 vs. 227. Subgroup age (<6 ys: 12 vs. 25); 6–<20 ys: 63 vs. 62; 20–<40 ys: 82 vs. 73; ≥40 ys: 11 vs. 67. Subgroup PMN <0.2 G/L: 70 vs. 94; ≥0.2 G/L: 98 vs. 133	0 vs. 34 (15)	0 vs. 0	
9	Fouladi 2000 [39] ‡	Toronto, Canada (1)	1987–1997	21 vs. 20	0 vs. 0	0 vs. 0	–
10	Führer 1998 [40]	SAA 94, Germany (37)	1993–1997	28 vs. 86	2 (7) vs. 7 (8)	0 vs. 0	4.3 vs. 3.0
11	Führer 2005 [41]	Germany (53)	1993–2001	Subgroup PMN <0.2 G/L: 40 vs. 97; <0.2–<0.5 G/L: 27 vs. 49	0 vs. 0	0 vs. 0	–
12	Ghavamzadeh 2004 [42]	Tehran, Iran (1)	1990–2001	29 vs. 24	0 vs. 0	0 vs. 0	–
13	Gillio 1997 [43]	New York, USA (1)	1983–1992	25 vs. 23. Subgroup year 1983–1987: not extractable; 1988–1992: not extractable	0 vs. 0	0 vs. 0	–
14	Gluckman 1979 [44] §	Paris, France (1)	unclear	37 vs. 28	0 vs. 0	0 vs. 0	6.8 vs. 7.2
15	Halperin 1989 [45]	Toronto, Canada (1)	1977–1987	14 vs. 12	0 vs. 0	0 vs. 0	–
16	Kahn 2002 [46]	Kansas City, USA (1)	1977–1999	15 vs. 16	0 vs. 0	0 vs. 0	6.0 vs. 2.0
17	Kim 2003 [47]	Seoul, Korea (1)	1990–1999	22 vs. 74	0 vs. 0	0 vs. 0	0.4 vs. 4.4
18	Kojima 2000 [48] ∥	Nagoya, Japan (2)	1984–1998	37 vs. 63	7 (19) vs. 14 (22)	0 vs. 0	–
19	Lawlor 1997 [49]	Vancouver, Canada (1)	1982–1994	9 vs. 18	0 vs. 0	0 vs. 0	3.0 vs. 2.7
20	Locasciulli 1990 [50]	EBMT, Europe (29)	1970–1988	171 vs. 133	0 vs. 0	23 (13) vs. 0	–
21	Locasciulli 2007 [51]	EBMT, Europe (257)	1991–2002	1567 vs. 912. Subgroup year (HSCT among patients with MSD) 1991–1996: 614 vs. 608; 1997–2002: 550 vs. 304. Subgroup age (IST among patients with PMN ≥0.2 G/L (vs. PMN <0.2 G/L)); <16 ys: 607 vs. 129 (vs. 175); ≥16 ys: 960 vs. 442 (vs. 141)	1183 (75) vs. 368 (40)	197 (13) vs. 0	4.6 vs. 4.7
22	Paquette 1995 [52]	Los Angeles, USA (1)	1977–1989	Subgroup year 1977–1983: 37 vs. 40; 1984–1989: 18 vs. 16	0 vs. 0	0 vs. 3 (5)	2.4 vs. 4.5
23	Pitcher 1999 [53] ¶	London, UK (1)	1973–1996	Subgroup year 1973–1988: unclear; 1989–1996: unclear	unclear	unclear	–
24	Tzeng 1989 [54]	Taipei, ROC (1)	1985–1988	9 vs. 7	0 vs. 0	0 vs. 0	–
25	Viollier 2005 [55] **	Basel, Switzerland (1)	1976–1999	52 vs. 155	14 (27) vs. 50 (32)	0 vs. 0	2.0 vs. 0.5
26	Werner 1989 [56] ††	Cincinnati, USA (2)	1981–1986	6 vs. 9	0 vs. 2 (22)	0 vs. 0	11.5 vs. 11.3

*other than SAA: MAA or not reported degree of severity.

† other than first-line MRD: second-line HSCT, first-line mismatched related HSCT, first-line- unrelated HSCT.

‡ Fouladi 2000: 5 patients in the IST group received second-line HLA-matched unrelated donor transplantation after failed IST.

§ Gluckman 1979: 5 patients of the IST group received second-line HLA-identical sibling donor transplantation after failed IST; 2 patients with Fanconi anemia and 2 patients with paroxysmal nocturnal hemoglobinemia were included.

∥ Kojima 2000: 11 patients in the IST group received second-line unrelated donor transplantation after failed IST.

¶ Pitcher 1999: outcome from 1973–1988 has been reported by Webb 1991 [69]; number of patients other than SAA estimated.

**Viollier 2005: Follow-up of Nissen 1999 [70], Speck 1994 [71], Tichelli 1988 [72], Speck 1986 [73], Speck 1984 [74], Speck 1983 [75], Speck 1981 [76], Speck 1980 [77], Speck 1977 [78]; 8 patients of the IST group received HSCT.

†† Werner 1989: 1 patient in the IST group received second-line haploidentical transplantation after failed IST.

Abbreviations: HLA: human leukocyte antigen; IST immunosuppressive therapy; PMN: polymorphonuclear neutrophil granulocytes; ROC: Republic of China; UK: United Kingdom of Great Britain and Nothern Ireland; USA: United States of America.

### Baseline data

An overview of characteristics of 7,955 analyzed patients in 26 studies is presented in Table 3. Median age (HSCT vs. IST: 9–24 vs. 5–55 years) was reported in 15 of 26 studies and the difference between groups was not statistically different (sign test: p = 0.119). Gender (HSCT vs. IST: 44–76 vs. 43–78 percent males) was reported in 19 of 26 studies and median of the time from diagnosis to treatment (HSCT vs. IST: 27–300 vs. 12–150 days) was reported in 15 of 26 studies.

**Table 3:** Patients' characteristics.

No	Study	Age; median years (range); HSCT vs. IST	Gender; N. males ∶ N. females (% males); HSCT vs. IST	Time interval from diagnosis to treatment; median days (range); HSCT vs. IST
1	Ahn 2003 [31]	(14–≥41) vs. (14–≥41)	33 ∶ 31 (57) vs. 72 ∶ 84 (46)	–
2	Arranz 1994 [32]	24 (12–46) vs. 38 (8–71)	–	50 (8–1145) vs. 60 (2–1889)
3	Bacigalupo 1988 [33]	(0–50) vs. (0–50)	135 ∶ 82 (62) vs. 162 ∶ 126 (56)	(0–>90) vs. (0–>90)
4	Bacigalupo 2000 [34]	–	–	90 vs. 35
5	Bayever 1984 [35]	17 (2–24) vs. 15 (1–23)	23 ∶ 12 (66) vs. 15 ∶ 7 (68)	60 (9–2520) vs. 58 (8–2669)
6	Champlin 1984 [36]	17 (1–44) vs. 31 (1–76)	43 ∶ 18 (71) vs. 41 ∶ 28 (59)	60 (7–1440) vs. 64 (3–2671)
7	De Planque 1990 [37]	27 (13–85) across groups	42 ∶ 40 across groups	–
8	Doney 1997 [38]	22 (2–53) vs. 25 (1–74)	101 ∶ 67 (60) vs. 102 ∶ 125 (45)	30 (6–6822) vs. 42 (3–4590)
9	Fouladi 2000 [39]	9 (2–16) vs. 10 (1–17)	16 ∶ 5 (76) vs. 11 ∶ 9 (55)	54 (18–165) vs. 12 (0–60)
10	Führer 1998 [40]	10 (2–16) vs. 9 (1–15)	12 ∶ 16 (43) vs. 53 ∶ 33 (62)	49 (18–272) vs. 23 (3–168)
11	Führer 2005 [41]	9 (1–17) across groups	125 ∶ 88 across groups	27 (1–268) across groups
12	Ghavamzadeh 2004 [42]	19 vs. 25 mean	19 ∶ 10 (63) vs. 18 ∶ 6 (75)	–
13	Gillio 1997 [43]	12 (2–19) vs. 14 (1–20)	14 ∶ 11 (56) vs. 18 ∶ 5 (78)	27 (5–2124) vs. 35 (10–4383)
14	Gluckman 1979 [44]	19 (3–31) vs. 21 (4–56)	23 ∶ 14 (62) vs. 15 ∶ 13 (54)	90 (8–2520) vs. 120 (15–1152)
15	Halperin 1989 [45]	8 (1–18) across groups	21 ∶ 15 across groups	27 (15–120) vs. 30 (6–180)
16	Kahn 2002 [46]	22 (6–59) vs. 55 (9–78)	8 ∶ 7 (53) vs. 10 ∶ 6 (63)	38 (14–866) vs. 12 (2–183)
17	Kim 2003 [47]	22 (14–43) vs. 34 (15–75)	16 ∶ 6 (73) vs. 37 ∶ 37 (50)	300 (30–3540) vs. 150 (30–10920)
18	Kojima 2000 [48]	10 (0–16) vs. 9 (1–17)	18 ∶ 19 (49) vs. 31 ∶ 32 (49)	38 (20–2040) vs. 24 (12–2490)
19	Lawlor 1997 [49]	13 (4–17) vs. 7 (2–14)	4 ∶ 5 (44) vs. 9 ∶ 9 (50)	–
20	Locasciulli 1990 [50]	(0–15) vs. (0–15)	97 ∶ 74 (57) vs. 71 ∶ 62 (53)	(0–>90) vs. (0–>90)
21	Locasciulli 2007 [51]	19 (1–67) vs. 24 (1–94)	959 ∶ 605 (61) vs. 520 ∶ 390 (57)	81 (1–3661) vs. 23 (1–1375)
22	Paquette 1995 [52]	(16–>30) vs. (16–>30)	–	–
23	Pitcher 1999 [53]	(1–14) across groups	40 ∶ 35 across groups	–
24	Tzeng 1989 [54]	20 (10–35) vs. 27 (19–56)	5 ∶ 4 (56) vs. 3 ∶ 4 (43)	–
25	Viollier 2005 [55]	19 (2–55) vs. 23 (2–74)	27 ∶ 25 (52) vs. 85 ∶ 70 (55)	51 (6–420) vs. 36 (1–11340)
26	Werner 1989 [56]	(3–15) vs. (1–16)	4 ∶ 2 (67) vs. 5 ∶ 4 (56)	(7–56) vs. (7–406)

–: information not extractable from publication.

Abbreviations: HSCT: hematopoietic stem cell transplantation; IST: immunosuppressive therapy.

An overview of the treatment characteristics is presented in Table 4. Conditioning irradiation was reported in 22 of 26 studies (0%–100%) and ATG or ALG was used in 9 studies (2%–100%) in the HSCT arms. In the IST arms, ATG or ALG (8%–100%) and CSA (0%–100%) was reported in 25 of 26 studies. IST was composed of ATG or ALG in 25 studies and was combined with CSA in 13 studies.

**Table 4:** Treatment characteristics.

No	Study	HSCT; N. treated/N. total (%)		IST; N. treated/N. total (%)	
		Irradiation*	ATG or ALG	ATG or ALG	CSA
1	Ahn 2003 [31]	6/64 (9)	53/64 (83)	148/156 (95)	67/156 (43)
2	Arranz 1994 [32]	20/21 (95)	–	29/29 (100)	0
3	Bacigalupo 1988 [33]	110/218 (50)	–	291/291 (100)	0
4	Bacigalupo 2000 [34]	433/1759 (25)	28/1759 (2)	–	–
5	Bayever 1984 [35]	35/35 (100)	0	22/22 (100)	0
6	Champlin 1984 [36]	59/61 (97)	0	69/69 (100)	0
7	De Planque 1990 [37]	15/19 (79)	1/19 (5)	83/83 (100)	0
8	Doney 1997 [38]	0	21/168 (24)	225/227 (99)	1/227 (1)
9	Fouladi 2000 [39]	12/21 (57)	5/21 (24)	20/20 (100)	19/20 (95)
10	Führer 1998 [40]	–	28/28 (100)	86/86 (100)	86/86 (100)
11	Führer 2005 [41]	0	0	VSAA: 97/97 (100)	VSAA: 97/97 (100)
				SAA: 49/49 (100)	SAA: 49/49 (100)
12	Ghavamzadeh 2004 [42]	0	29/29 (100)	2/24 (8)	24/24 (100)
13	Gillio 1997 [43]	17/25 (68)	3/25 (12)	23/23 (100)	0
14	Gluckman 1979 [44]	10/37 (27)	9/37 (24)	28/28 (100)	0
15	Halperin 1989 [45]	14/14 (100)	14/14 (100)	12/12 (100)	0
16	Kahn 2002 [46]	8/15 (53)	0	16/16 (100)	16/16 (100)
17	Kim 2003 [47]	17/22 (77)	2/22 (9)	74/74 (100)	17/74 (23)
18	Kojima 2000 [48]	26/37 (70)	9/37 (24)	27/63 (43)	2/63 (3)
19	Lawlor 1997 [49]	2/9 (22)	1/9 (11)	18/18 (100)	15/18 (83)
20	Locasciulli 1990 [50]	59/171 (35)	34/171 (20)	133/133 (100)	0
21	Locasciulli 2007 [51]	282/1567 (18)	319/1567 (20)	495/912 (54)	846/912 (93)
22	Paquette 1995 [52]	–	–	1977–1983: 40/40 (100)	0
				1984–1989: 16/16 (100)	
23	Pitcher 1999 [53]	–	–	1973–1988: 18/18 (100)	1973–1988: 0
				1989–1996: 25/25 (100)	1989–1996: 14/25 (56)
24	Tzeng 1989 [54]	9/9 (100)	0	7/7 (100)	0
25	Viollier 2005 [55]	–	–	155/155 (100)	0
26	Werner 1989 [56]	2/6 (33)	–	8/9 (89)	2/9 (22)

*Irradiation: conditioning irradiation: thoraco-abdominal irradiation, total body irradiation, or total lymphoid irradiation.

Abbreviations: ALG: anti-lymphocyte globulin; ATG: anti-thymocyte globulin; CSA: cyclosporine A; HSCT: hematopoietic stem cell transplantation; IST: immunosuppressive therapy.

### Primary outcome: overall survival

An overview of the overall survival is presented in Table 5. 5-year overall survival was reported in 17 studies (HSCT vs. IST: 32%–98% vs. 37%–92%). 5 studies reported a statistically significant difference of survival functions in favor of HSCT [38], [47], [48], [50], [51] and 1 study in favor of IST [46]. 9 studies [31], [33], [35], [40], [42], [43], [49], [54], [55] did not find a statistically significant difference of survival functions and 11 studies did not report a significance test.

**Table 5:** Five-year overall survival.

No	Study	Total		Subgroups		Comments
		Meta-analysis	HSCT vs. IST in % (p-value§)	Meta-analysis	HSCT vs. IST in % (p-value§)	
1	Ahn 2003 [31]	Yes	79* vs. 72* (0.83)	–	–	–
2	Arranz 1994 [32]	Yes	71† vs. 62† (–)	–	3 years (20–40 ys: 63† vs. 47†)	–
3	Bacigalupo 1988 [33]	Yes	63† vs. 61† (0.1)	AgePMN	Subgroup age+PMN: <20 ys + <0.2 G/L: 64† vs. 38† (0.01); <20 ys + ≥0.2 G/L: 58† vs. 62† (0.1); 4 years (≥20 ys + <0.2 G/L: 62† vs. 82† (0.002)); 4 years (≥20 ys + ≥0.2 G/L: 44† vs. 43† (0.06))	Younger patients (<20 ys) had a better survival with HSCT than with IST, *p* = 0.01. This result remained stable for patients with <0.2 G/L (*p* = 0.01) but not with ≥0.2 G/L (*p* = 0.1). Patients aged ≥20 ys had a better survival with IST than with HSCT, p = 0.0008. This result remained stable for patients with ≥0.2 G/L (*p* = 0.002) but not with <0.2 G/L (*p* = 0.06).
4	Bacigalupo 2000 [34]	–	–	Year	Subgroup IST = ALG: 1976–1989: 56† vs. 50† (–); 1990–1998: 80† vs. 63† (–); Subgroup IST = ALG+CSA; 1976–1989: 56† vs. 64† (–); 1990–1998: 80† vs. 83† (–)	HSCT: Age 1990–1998: <17 ys (77) vs. 17–40 ys (68) vs. ≥41 ys (54), *p* = 0.001. The outcome among transplanted patients was better with younger age. Survival has improved in all age groups over time and the effect of age remained. Patients receiving radiation had no survival advantage but a higher incidence of chronic GVHD and malignant disease. IST: Age (unclear treatment period): <17 (64) vs. 17–40 (71) vs. ≥41 (59). There was no age effect on outcome of IST patients.
5	Bayever 1984 [35]	Yes	4 years(72* vs. 45* (0.18))	–	–	–
6	Champlin 1984 [36]	–	61† vs. 56† (–)	Age	Subgroup age: 4 years (<20: 79† vs. 37* (0.02)); 20–<35: 41† vs. 68* (not sign.); 2 years (≥35: 20* vs. 60* (0.02))	Survival function reported only for subgroups
7	De Planque 1990 [37]	Yes	32† vs. 63† (–)	–	–	–
8	Doney 1997 [38]	Yes	72* vs. 48*(0.01 favors HSCT)	AgePMN	Subgroup age: <6 ys: 100* vs. 55* (0.006); 6–<20 ys: 78* vs. 55* (0.001); 20–<40 ys: 69* vs. 57* (0.04); ≥40 ys: 37* vs. 39* (0.2). Subgroup PMN: <0.2 G/L: 74* vs. 38* (–); ≥0.2 G/L: 74* vs. 58* (–)	–
9	Fouladi 2000 [39]	Yes	95* vs. 65*	–	–	–
10	Führer 1998 [40]	Yes	4 years(84† vs. 87† (0.43))	–	–	–
11	Führer 2005 [41]	–		–	Subgroup PMN: <0.2 G/L: 89† vs. 93† (–); ≥0.2 G/L: 96† vs. 81† (–)	Survival function reported only for IST group; significant difference within the IST group, p<0.001.
12	Ghavamzadeh 2004 [42]	Yes	67† vs. 37† (0.3)	–	–	–
13	Gillio 1997 [43]	Yes	83* vs. 86* (0.98)	–	Subgroup year: 1983–1987: 72* vs. 85* (not sign.); 1988–1992: 100* vs. 89* (not sign.)	Number of patients not reported for treatment periods.
14	Gluckman 1979 [44]	–	–	–	–	–
15	Halperin 1989 [45]	Yes	78* vs. 43* (–)	–	–	–
16	Kahn 2002 [46]	Yes	33* vs. 70*(0.047 favors IST)	–	–	–
17	Kim 2003 [47]	Yes	95† vs. 70†(0.04 favors HSCT)	–	–	–
18	Kojima 2000 [48]	Yes	98* vs. 80*(0.004 favors HSCT)	–	–	–
19	Lawlor 1997 [49]	Yes	75† vs. 92† (0.15)	–	–	–
20	Locasciulli 1990 [50]	Yes	64* vs. 52*(0.002 favors HSCT)	–	–	–
21	Locasciulli 2007 [51]	Yes	74* vs. 75*(0.002 favors HSCT)	AgeYearPMN	Subgroup year (HSCT among patients with MSD): 1991–1996: 75* vs. 76* (–); 1997–2002: 80* vs. 73* (–). Subgroup age (IST among patients with PMN: ≥0.2 (vs. <0.2 G/L)); <16 ys: 76* vs. 78* (vs. 83*) (–); ≥16 ys: 64* vs. 73* (vs. 65*) (–)	HSCT: The outcome among patients with MSD has improved over time (p = 0.03). IST: The outcome among patients with <0.2 G/L was better in children (<16 ys) than in adults (≥16 ys), *p* = 0.0002. The outcome among patients with ≥0.2 G/L was similar between age groups, *p* = 0.2.
22	Paquette 1995 [52]	–	–	Year	1977–1983: 42* vs. 54* (not sign.); 1984–1989: 72* vs. 45* (0.25)	–
23	Pitcher 1999 [53]	–	–	–	1973–1988: 68* vs. 44* (–); 1989–1996: 92* vs. 86* (–)	Unclear number of patients
24	Tzeng 1989 [54]	Yes	2 years (75† vs. 43† (>0.1))	–	–	–
25	Viollier 2005 [55]	Yes	57* vs. 68* (0.2)	–	–	Survival, event-free survival, and quality-adjusted time without symptoms and toxicity are similar between HSCT and IST. There were differences in terms of mean duration in years of some health states (HSCT vs. IST): Treatment-related toxicity 0.27 vs. 0.36, *p*<0.001; transfusion dependency 0.1 vs. 0.66, *p*<0.001; secondary clonal disorder 0.04 vs. 0.68, *p*<0.001; extensive chronic GVHD 0.96 vs. 0, *p*<0.023
26	Werner 1989 [56]	–	–	–	–	–

–: information not extractable from the publication.

*5-year overall survival point estimate deduced from Kaplan Meier curve.

† 5-year overall survival point estimate extracted from text.

‡ Kahn 2002: 5-year overall survival 33% vs. 78% (text), 33% vs. 70% (figure).

§ p-value of log-rank test.

Abbreviations: CI: 95% confidence interval, lower-upper limit; HSCT: hematopoietic stem cell transplantation; IST: immunosuppressive therapy; PMN: polymorphonuclear neutrophil granulocytes.

**Figure 2:**
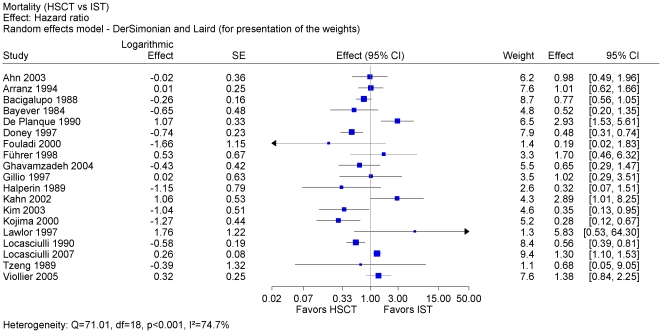
Meta-analysis of all studies with available data. Meta-analysis of overall survival using hazard-ratio as effect measure after first-line HSCT vs. first-line IST. Pooled estimate not justified because of considerable heterogeneity and of not sufficient external validity. Abbreviations: HSCT: hematopoietic stem cell transplantation; IST: immunosuppressive therapy; SAA: severe aplastic anemia; SE: standard error.

We included 19 studies (4,855 patients) [31], [32], [33], [35], [37], [38], [39], [40], [42], [43], [45], [46], [47], [48], [49], [50], [51], [54], [55] in a meta-analysis on overall survival, which provided summary statistics required for estimating the hazard ratio. The pooled hazard ratio was statistically not significant and was characterized by a considerable heterogeneity indicated by an I^2^ value of 75% (Figure 2). A pooled estimate was not justified.

### Risk of bias

Risk of bias within studies was high for 21 of 26 included studies mainly due to flaws of study design, assignment of patients to treatment groups, and missing control for confounding (Table 6). In 7 studies, an upper age limit was applied as additional assignment criteria.

**Table 6:** Risk of bias.

No	Included studies	Prospective design	Concurrent control	No other assignment criteria than MRD	Control for confounding factors*	No other risk of bias factors†	Risk of bias‡
1	Ahn 2003 [31]	no	YES	YES	YES	YES	LOW
2	Arranz 1994 [32]	no	YES	no (age 40)§	no	YES	high
3	Bacigalupo 1988 [33]	no	YES	YES	YES	YES	LOW
4	Bacigalupo 2000 [34]	no	YES	YES	YES	YES	LOW
5	Bayever 1984 [35]	no	YES	YES	no	YES	high
6	Champlin 1984 [36]	no	YES	no (age 45)§	no	no (selection unclear)	high
7	De Planque 1990 [37]	no	YES	no (second-line)	no	YES	high
8	Doney 1997 [38]	no	YES	no (age 55)§	no	YES	high
9	Fouladi 2000 [39]	no	YES	YES	no	YES	high
10	Führer 1998 [40]	no	YES	YES	no	YES	high
11	Führer 2005 [41]	YES	YES	YES	no	YES	high
12	Ghavamzadeh 2004 [42]	no	YES	no (age 45)§	no	YES	high
13	Gillio 1997 [43]	no	YES	YES	no	YES	high
14	Gluckman 1979 [44]	no	YES	YES	no	no (5 in both groups)	high
15	Halperin 1989 [45]	no	YES	YES	no	YES	high
16	Kahn 2002 [46]	no	no	no (age 40)§	no	YES	high
17	Kim 2003 [47]	no	YES	no (age 50)§	no	YES	high
18	Kojima 2000 [48]	no	YES	YES	no	YES	high
19	Lawlor 1997 [49]	no	YES	YES	no	YES	high
20	Locasciulli 1990 [50]	no	YES	YES	YES	YES	LOW
21	Locasciulli 2007 [51]	no	YES	no (alternate donor)	no	YES	high
22	Paquette 1995 [52]	no	YES	YES	no	no (selection unclear)	high
23	Pitcher 1999 [53]	no	YES	YES	no	YES	high
24	Tzeng 1989 [54]	no	YES	YES	no	YES	high
25	Viollier 2005 [55]	YES	YES	no (age 40)§	no	YES	high
26	Werner 1989 [56]	no	YES	YES	no	YES	high

*Control for confounding factors; no: no adjusted analysis.

† No other risk of bias factors; no: selection of patients unclear; except Gluckman 1979: no: 5 patients with failed first-line IST followed by second-line HSCT were analyzed in both treatment groups.

‡ Risk of bias: LOW required concurrent control group (YES), control for confounding factors (YES), and no other risk of bias factors (YES).

§ Upper age limit in years.

Abbreviations: HSCT: hematopoietic stem cell transplantation; IST immunosuppressive therapy; MRD: HLA-matched related donor; PMN: polymorphonuclear neutrophil granulocytes.

**Figure 3:**
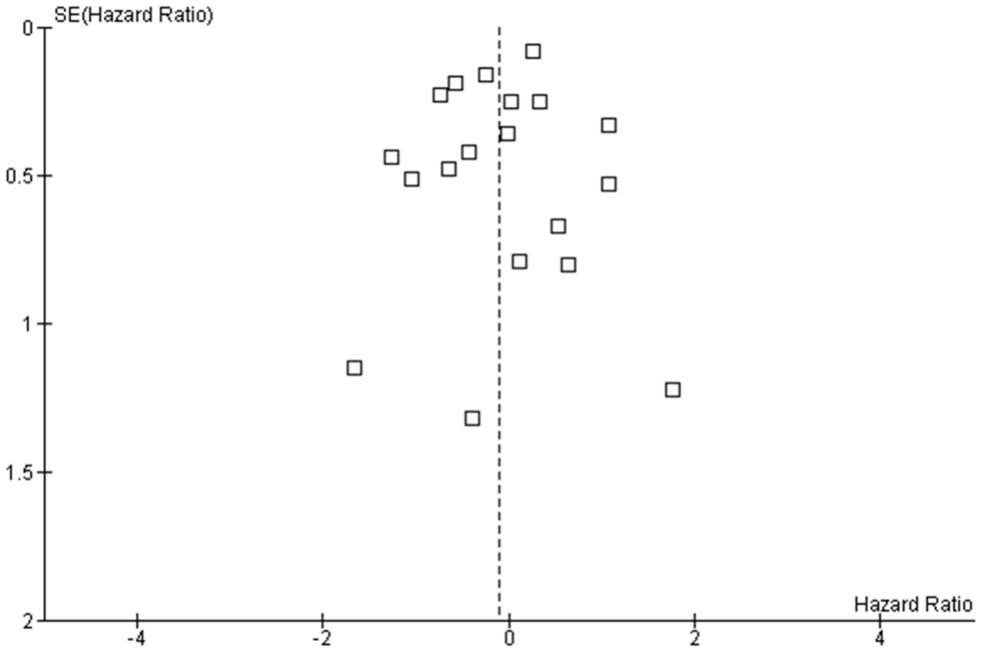
Funnel plot using data from **Figure 3**. Distribution of estimates can be regarded as funnel-shaped and compatible with a moderate publication bias. Abbreviations: SE: standard error.

The funnel plot of 19 studies included in meta-analysis shows no asymmetry (Figure 3), which may be consistent with absent publication bias. In 7 of 26 included studies, data of the primary outcome were not sufficiently reported to be included in the main meta-analysis.

### Subgroup and sensitivity analysis

The difference between the estimates was not statistically significant in both approaches and for all evaluated items, except for age, which was statistically significant in the evaluation of subgroups from individual studies and not statistically significant in the sensitivity analysis (Table 7).

**Table 7:** Sensitivity analysis.

Characteristic	Type of analysis	Subgroups	Included studies	Statistics*
Age	Sensitivity analysis	Median age in HSCT or IST group ≥18 years vs. <18 years	[32], [38], [46], [47], [51], [54], [55] vs. [35], [39], [40], [43], [48], [49]	*p* = 0.449
Age	Subgroups in individual studies	Advanced vs. young age (various definitions)	[33], [36], [38], [51]	*p* = 0.040
Year of treatment	Sensitivity analysis	Center year of observation period (HSCT or IST) ≥1995 vs. <1995	[31], [42], [50], [51] vs. [32], [33], [35], [37], [38], [39], [40], [43], [45], [46], [47], [48], [49], [54], [55]	*p* = 0.945
Year of treatment	Subgroups in individual studies	Recent vs. early year of treatment (various definitions)	[33], [51], [52]	*p* = 0.884
Severity of disease	Subgroups in individual studies	high PMN (SAA without VSAA) vs. low PMN (VSAA)	[33], [38], [51]	*p* = 0.086
Study population	Sensitivity analysis	Number of patients per study ≥100 vs. <100	[31], [33], [38], [40], [48], [50], [51], [55] vs. [32], [35], [37], [39], [42], [43], [45], [46], [47], [49], [54]	*p* = 0.604
Time interval HSCT	Sensitivity analysis	Median interval from diagnosis to HSCT ≥50 days vs. <50 days	[32], [35], [39], [47], [51], [55] vs. [38], [40], [43], [45], [46], [48]	*p* = 0.809
Time interval IST	Sensitivity analysis	Median interval from diagnosis to IST ≥50 days vs. <50 days	[32], [35], [47] vs. [38], [39], [40], [43], [45], [46], [48], [50], [55]	*p* = 0.530
Location	Sensitivity analysis	Multicenter study vs. single center	[31], [33], [40], [48], [50], [51] vs. [32], [35], [37], [38], [39], [42], [43], [45], [46], [47], [49], [54], [55]	*p* = 0.474

*Statistics: test of interaction.

### Factors for improved overall survival

We evaluated patient characteristics that were evaluated in multivariate regression analyses (Table 8). 11 studies reported statistically significant factors for improved survival and 15 studies did not. Frequency of reporting the main factors, that is, age, year of transplant and type of IST is presented in Table 9.

**Table 8:** Factors influencing overall survival with statistical significance.

No	Included studies	Characteristic	Favors HSCT	Favors IST
1	Ahn 2003 [31]	Not reported	n.a.	n.a.
2	Arranz 1994 [32]	Not reported	n.a.	n.a.
3	Bacigalupo 1988 [33]	Age (years)	<20	≥20
		PMN (G/L)	<0.2	0.2–<0.5
4	Bacigalupo 2000 [34]	Age (years)	<17; <41	No effect
		Year of transplant	≥1990	Not reported
		Type of IST	n.a.	ALG+CSA (vs. ALG or CSA alone)
5	Bayever 1984 [35]	Not reported	n.a.	n.a.
6	Champlin 1984 [36]	Age (years)	<20	≥20
7	De Planque 1990 [37]	Age (years)	No effect	<45
		PMN (G/L)	No effect	≥0.2
8	Doney 1997 [38]	Age (years)	<40	–
		PMN (G/L)	No effect	≥0.2
9	Fouladi 2000 [39]	PMN (G/L)	No effect	≥0.2
10	Führer 1998 [40]	Not reported	n.a.	n.a.
11	Führer 2005 [41]	PMN (G/L)	–	<0.2 (among patients aged 0 to 17 years)
12	Ghavamzadeh 2004 [42]	Not reported	n.a.	n.a.
13	Gillio 1997 [43]	Not reported	n.a.	n.a.
14	Gluckman 1979 [44]	Not reported	n.a.	n.a.
15	Halperin 1989 [45]	Not reported	n.a.	n.a.
16	Kahn 2002 [46]	Not reported	n.a.	n.a.
17	Kim 2003 [47]	Not reported	n.a.	n.a.
18	Kojima 2000 [48]	Not reported	n.a.	n.a.
19	Lawlor 1997 [49]	Not reported	n.a.	n.a.
20	Locasciulli 1990 [50]	Year of transplant	≥1981	No effect
		Age (years)	No effect	≥6 (among patients with PMN <0.2 G/L)
		PMN (G/L)	No effect	≥0.2
21	Locasciulli 2007 [51]	Age (years)	<16	<16 (among patients with PMN <0.2 G/L)
		Year of treatment	≥1997	No effect
		Type of IST	n.a.	ALG+CSA
		PMN (G/L)	Not reported	No effect
22	Paquette 1995 [52]	Not reported	n.a.	n.a.
23	Pitcher 1999 [53]	Year of transplant	≥1989	≥1989
24	Tzeng 1989 [54]	Not reported	n.a.	n.a.
25	Viollier 2005 [55]	Quality-adjusted time without symptoms and toxicity	Treatment-related toxicity; transfusion dependency; secondary clonal disorder	Extensive chronic GVHD
26	Werner 1989 [56]	Not reported	n.a.	n.a.

–: information not extractable from the publication.

*5-year overall survival point estimate deduced from Kaplan Meier curve.

† 5-year overall survival point estimate extracted from text.

‡ Kahn 2002: 5-year overall survival 33% vs. 78% (text), 33% vs. 70% (figure).

§ *p*-value of log-rank test.

Abbreviations: ALG: anti-lymphocyte globulin; CSA: cyclosporine A; GVHD: graft-versus-host disease; HSCT: hematopoietic stem cell transplantation; IST: immunosuppressive therapy; n.a.: not applicable; PMN: polymorphonuclear neutrophil granulocytes.

**Table 9:** Frequency of reporting statistically significant factors of improved survival.

Characteristic	HSCT	IST
Age (years): young vs. advanced	5 [34], [35], [37], [39], [52]	2 [38], [52]
Age (years): advanced vs. young	0	3 [34], [37], [51]
PMN (G/L): <0.2 vs. 0.2 to <0.5	1 [34]	1 [42]
PMN (G/L): 0.2 to <0.5 vs. <0.2	0	5 [34], [38]–[40], [51]
Year of treatment: recent vs. earlier period	4 [35], [51], [52], [54]	1 [54]
Type of IST: ALG+CSA vs. ALG	n.a.	2 [35], [52]

Abbreviations: ALG: anti-lymphocyte globulin; CSA: cyclosporine A; HSCT: hematopoietic stem cell transplantation; IST: immunosuppressive therapy; n.a.: not applicable; PMN: polymorphonuclear neutrophil granulocytes.

Young age was identified as a statistically significant factor for improved overall survival for patients in the HSCT group in 5 studies. The results for patients in the IST group were less clear because advanced age was a favorable factor in 3 studies and young age a favorable factor in 2 studies. The actual age limit, which was used to dichotomize participants into young vs. advanced age varied across studies. Level of polymorphonuclear neutrophilic granulocytes (PMN) between 0.2 G/L and 0.5 G/L was found to be an important factor for improved survival after IST in 5 studies. Only 1 study found an advantage with less than 0.2 G/L in patients who were younger than 17 years. Recent year of treatment was an important factor for improved survival after HSCT in 4 studies and after IST in 1 study. Improvement of outcome over time was accompanied with treatment refinement, such as combination of methotrexate with CSA for GVHD prophylaxis and reduction of conditioning irradiation. Another example of improvement of outcome with treatment refinement is the combination of ALG and CSA instead of monotherapy of ALG. This combination was identified as factor for improved survival in 2 studies.

### Secondary outcomes

An overview of graft failure, GVHD, no response, and overall mortality is presented in Table 10. Graft failure (reported in 15 studies) ranged from 1% to 43%, acute GVHD grade II to IV (reported in 16 studies) ranged from 0% to 88%, and extensive chronic GVHD (reported in 10 studies) ranged from 3 to 27%. No response to IST (extractable from 14 studies) ranged from 6% to 71%. Overall mortality was reported in 23 studies (HSCT vs. IST: 3%–67% vs. 9%–58%).

**Table 10:** Adverse events: graft failure, GVHD, no response, overall mortality.

No	Study	HSCT; N. affected/N. evaluable (%)			IST; N. affected/N. evaluable (%)	HSCT vs. IST; N. affected/N. evaluable (%)
		Any graft failure	Acute GVHD grade II–IV	Extensive chronic GVHD	No response	Overall mortality
1	Ahn 2003 [31]	11/61 (18)	20/50 (40)	12/50 (24)	–	13/64 (20) vs. 37/156 (24)
2	Arranz 1994 [32]	–	–	–	–	9/71 (13) vs. 8/62 (13)
3	Bacigalupo 1988 [33]	–	–	–	–	87/218 (40) vs. 75/291 (26)
4	Bacigalupo 2000 [34]	211/1759 (12)	228/1759 (13)	176/1759 (10)		611/1759 (35) vs. 540/1592 (34)
5	Bayever 1984 [35]	1/35 (3)	10/34 (29)	7/34 (21)	14/22 (64)	9/35 (26) vs. 9/22 (41)
6	Champlin 1984 [36]	3/61 (9)	25/58 (43)	9/58 (16) any type	33/69 (48)	23/61 (38) vs. 26/69 (38)
7	De Planque 1990 [37]	3/19 (16)	14/16 (88)	3/11 (27)	16/82 (20)	8/19 (42) vs. 25/63 (40)
8	Doney 1997 [38]	18/168 (11)	46/150 (31)	26/150 (17)	122/227 (54)	50/168 (30) vs. 130/227 (57)
9	Fouladi 2000 [39]	1/21 (5)	3/20 (15)	1/20 (5)	–	1/21 (5) vs. 6/20 (30)
10	Führer 1998 [40]	–	–	–	–	4/28 (14) vs. 8/86 (9)
11	Führer 2005 [41]	–	–	–	–	–
12	Ghavamzadeh 2004 [42]	–	14/29 (48)	–	14/24 (58)	9/29 (31) vs. 14/24 (58)
13	Gillio 1997 [43]	1/25 (4)	2/25 (8) III–IV	2/25 (8)	13/23 (57)	5/25 (20) vs. 5/23 (22)
14	Gluckman 1979 [44]	16/37 (43)	8/37 (22)	–	20/28 (71)	20/37 (54) vs. 14/29 (48)
15	Halperin 1989 [45]	–	–	–	1/12 (8)	3/14 (21) vs. 7/12 (58)
16	Kahn 2002 [46]	3/15 (20)	7/15 (47) any type	1/15 (7) any type	1/16 (6)	10/15 (67) vs. 5/16 (31)
17	Kim 2003 [47]	1/22 (5)	2/21 (10)	3/21 (14)	41/74 (55)	2/22 (9) vs. 20/74 (27)
18	Kojima 2000 [48]	1/37 (3)	2/36 (6)	1/36 (3)	31/63 (49)	1/37 (3) vs. 21/63 (33)
19	Lawlor 1997 [49]	1/9 (11)	5/9 (56)	3/9 (33) any type	5/18 (28)	2/9 (22) vs. 2/18 (11)
20	Locasciulli 1990 [50]	–	–	–	–	58/171 (34) vs. 57/133 (44)
21	Locasciulli 2007 [51]	–	42/1567 (3) any	–	–	371/1567 (24) vs. 228/912 (25)
22	Paquette 1995 [52]	–	–	–	–	–
23	Pitcher 1999 [53]	–	–	–	–	–
24	Tzeng 1989 [54]	2/9 (22)	0/9 (0)	2/9 (22) any type	4/7 (57)	2/9 (22) vs. 4/7 (57)
25	Viollier 2005 [55]	5/52 (1)	–	12/52 (23)	41/155 (27)	24/52 (46) vs. 61/155 (39)
26	Werner 1989 [56]	–	–	–	–	0/6 (0) vs. 1/9 (11)

–: information extractable from publication.

*Bayever 1984: acute GVHD including interstitial pneumonia; chronic GVHD including moderate or severe types. Abbreviations: GVHD: graft-versus-host disease; HSCT: hematopoietic stem cell transplantation; IST: immunosuppressive therapy.

† Bacigalupo 2000: less acute GVHD with methotrexate plus cyclosporine A vs. methotrexate or cyclosporine A alone; incidence of acute GVHD III to IV has been reduced from 20% to 6% with time (*p*<0.00001), however, survial for patients with acute GVHD>I has not improved with time; incidence of extensive chronic GVHD has been reduced from 15% to 5% with time (*p*<0.00001), however, survival for patients with extensive chronic GVHD beyond day 100 after HSCT has not been improved with time.

Abbreviations: GVHD: graft-versus-host disease; HSCT: hematopoietic stem cell transplantation; IST: immunosuppressive therapy.

## Discussion

### Primary outcome

We found high risk of bias among 26 identified nonrandomized controlled studies. Considerable heterogeneity of 19 studies included in a meta-analysis of overall survival did not justify a pooled estimate. The aim to compare the primary outcome between treatment groups in an overall synthesis of available data was not achieved. We were able to identify statistically significant factors for improved overall survival reported in the studies. Young age (rather than advanced age) and recent (rather than earlier) year of treatment were associated with a better overall survival in the HSCT group. Advanced age (rather than young age), SAA without VSAA (rather than VSAA), and combination of ALG and CSA (rather than ALG alone) were associated with a better overall survival in the IST group. While pooling data on overall survival of all participating patients did not appear sensible, we conducted sensitivity meta-analysis of subgroups to explain heterogeneity and of subgroups reported in individual studies. Unfortunately, these evaluations were hampered by the fact that individual patient data were not available and instead of that we relied on published aggregate data if reported. It should be mentioned that ALG is no longer available.

Although young age was recognized as a major influence factor on overall survival, the appropriate definition for young age varies considerably across studies. For example, the cut-off for young vs. advanced age was 16 years in the study of Bacigalupo 2008 [16], 30 years in the study of Ljungman 2009 [59], and 40 years in the study of Marsh 2009 [9].

Bacigalupo 2008 [16] reported that the outcome has improved since 1996 for HSCT but not for IST. This result was supported by 4 studies whereas 1 study did find an improvement for the IST group as well. Several factors may have contributed to recent improvements for HSCT, such as detailed HLA-matching, less irradiation-based conditioning, less acute GVHD with a prophylaxis combination of methotrexate plus CSA instead of methotrexate alone [34], refinement of the type and dosage of conditioning drugs, and general advancement of medical and nursing clinical science.

We found that a combination therapy of ALG plus CSA favored overall survival in the IST group, underscoring that refinement of therapy obviously has also improved the outcome with IST. Gafter-Gvili 2008 [60] concluded in a systematic review and meta-analysis that combination of ALG plus CSA should be considered the gold standard for IST for patients with SAA.

6 studies [33], [37], [38], [39], [41], [50] evaluated whether the disease severity of VSAA (PMN <0.2 G/L) vs. SAA (without VSAA; PMN 0.2 to <0.5 G/L) had an impact on overall survival. 5 studies [33], [37], [38], [39], [50] consistently found that SAA without VSAA favored the outcome in IST group when compared to VSAA. 2 studies [39], [50] included only children and 3 studies [33], [37], [38] included also adults. These results may suggest that HSCT may be the preferred treatment option for patients with VSAA and that IST may be the preferred option for patients with SAA without VSAA. However, Führer 2005 [41] reported contradictory results that VSAA favored the outcome in IST group. The results may be relevant for children only because all analyzed patients were younger than 17 years of age. We did not find another study confirming the results, especially not in 2 studies mentioned above.

We believe that this is the first comprehensive systematic review and meta-analysis about studies comparing first-line HSCT versus first-line IST in patients with SAA.

### Secondary outcomes

Studies inconsistently reported adverse events and their frequencies varied significantly across studies. Graft failure was highest in early studies but could affect up to 18% of patients in recent studies. Rates for acute GVHD grade III to IV reached up to 40% and for extensive chronic GVHD reached up to 24% in recent studies. A considerable number of patients not responding to IST may indicate the importance of unrelated donor transplantation.

### Duplicate publication bias

Identical data may have been included twice in the present systematic review. We searched for follow-up papers of a single study to include the update version and exclude former presentations. Register analyses can provide results based on a large number of patients but registers may use data that may have been published previously by the contributing study centers. We identified 5 studies published on behalf of the EBMT [33], [34], [50], [51] which are probably based on overlapping data. Locasciulli 2007 [51] presented an update of the EBMT data and, therefore, investigated considerably more patients (2479 patients) than Locasciulli 1990 [50] (304 patients). The courses of the survival functions are clearly different between the 2 studies. In Locasciulli 1990 [50] (Figure 1 of the article), in the majority of the follow-up period the course of the Kaplan-Meier curve after HSCT is above that after IST (congruent in the first 12 months), indicating an advantage from HSCT. In Locasciulli 2007 [51] (Figure 1 of the article), in the majority of the follow-up period the course of the Kaplan-Meier curve after HSCT is below that after IST (in the first 60 months). Only at the very end of the follow-up period (from 100 to 120 months) is the HSCT curve above the IST curve. The difference between the 2 survival functions was assessed by a log-rank test. The authors stated that *10-year survival was significantly superior in patients treated with BMT than in those in whom immunosuppression was used (73% versus 68%, p = 0.002)*. On the contrary, we are convinced that the survival functions clearly show that IST was statistically significantly better than HSCT. Our interpretation of the results is supported by Linden 2007 [61], who addressed the specific problem of interpreting the crossing of the survival functions using Locasciulli 2007 [51] as an example. Locasciulli 1990 [50] clearly reported that only data from SAA patient were included. In contrast, Locasciulli 2007 [51] included a considerable proportion of patients with moderate aplastic anemia or with unknown severity of disease. A study population with different patients' characteristics with respect to disease severity might have contributed to the different results. Medical advancement after a time difference of 17 years between publication dates of both studies might have had a greater impact on improved survival after HSCT than on survival after IST.

### Outcome reporting bias

Outcome reporting bias [62] is defined as the selection of a subset of the originally recorded outcome variables for publication. Systematic reviews need to address the issue of missing outcome data because outcome reporting bias can affect their conclusions [63]. We identified considerable outcome reporting bias. A major flaw of all studies was the lack of statistical summary data such as standard error or confidence interval for point estimates and *p*-values of log-rank test. To pool as many studies as possible, we estimated the hazard ratio. The number of patients at risk was scarcely reported.

### Study publication bias

Study publication bias is defined as publication of research results depending on their results [64]. Funnel plots of both reported meta-analyses show moderate asymmetry and do not indicate considerable publication bias. The strengths of the present systematic review are the broadness of the search strategy and the comprehensiveness of the published data included. Nevertheless, there may be a slight possibility that an unknown number of studies were not registered and not published.

### Language bias

Results in English language articles could be different from those of articles written in other languages [65]. Non-English language articles require expensive translations to prevent selective outcome extraction and misinterpretation of results. Funding for translation was not provided and we excluded all non-English language articles, including German articles. Restricting the inclusion of studies to English articles may have little effect on summary treatment effect estimates [66], [67] and German language articles may not play a preeminent role in the dissemination of medical research [68].

### Internal validity

We identified a high risk of bias within all non-randomized controlled studies except for 4 studies. Assignment of patients to treatment groups was reported to be based on availability of MRD in all 26 studies, although, in 7 studies an upper age limit was applied as additional assignment criteria. This type of allocation has specific requirements, such as allelic vs. serologic typing, number of analyzed loci, time spent searching for donors, documentation of all individuals analyzed including families of IST patients, number of analyzed individuals per family, intent-to-treat analysis. However, these requirements were not reported in the included studies.

### Heterogeneity

We included as many controlled studies as possible in order to not miss any valuable outcome information. Consequently, we accepted studies that considered a considerable number of patients with moderate aplastic anemia or unknown disease severity. Pooling of data from patients with varying characteristics may have compromised the generalizability of results. Furthermore, important subgroups such as young and advanced age were confused in the meta-analysis.

In an attempt to reduce heterogeneity we strictly confined the meta-analysis to studies that clearly included at least 80% of patients with SAA and first-line treatment (data not shown). We found a moderate heterogeneity and a statistically significant pooled estimate that favored HSCT. Many studies including large and recently published ones were excluded. The result suggested a global preference for one treatment disregarding conditions other than the treatment that might have a determined impact on the outcome. We believe that this procedure would have introduced a study selection bias and a misleading conclusion and was therefore not pursued.

### Strengths and limitations of the present review

The strengths of this review are the broadness of the search strategy and the comprehensiveness of the published data included. Significant factors that may influence the survival of the patients were considered in the present systematic review. We estimated hazard ratios from published aggregate survival functions and did not use individual patient data. Subgroup analysis was not helpful to explain considerable heterogeneity found in meta-analysis. While the results of the meta-analysis may not be conclusive, they can provide useful summaries of the state of knowledge.

### Conclusions

Young age and recent year of treatment were identified as factors for improved survival in the transplant group. Advanced age, SAA without very severe aplastic anemia, and combination of anti-lymphocyte globulin with cyclosporine A were factors for improved survival in the immunosuppressive group. Considerable heterogeneity of non-randomized controlled studies did not justify a pooled estimate. Adverse events were inconsistently reported and varied significantly across studies.

## References

[1] ORD (2010). Office of Rare Diseases Terms: Aplastic anemia.

[2] Kaufman DW, Kelly JP, Issaragrisil S, Laporte JR, Anderson T (2006). Relative incidence of agranulocytosis and aplastic anemia.. Am J Hematol.

[3] Brodsky RA, Jones RJ (2005). Aplastic anaemia.. Lancet.

[4] Young NS, Calado RT, Scheinberg P (2006). Current concepts in the pathophysiology and treatment of aplastic anemia.. Blood.

[5] EBMT-AAWP (2000). Guidelines for treating of aplastic anemia.

[6] Ljungman P, Urbano-Ispizua A, Cavazzana-Calvo M, Demirer T, Dini G (2006). Allogeneic and autologous transplantation for haematological diseases, solid tumours and immune disorders: Definitions and current practice in Europe.. Bone Marrow Transplant.

[7] Marsh J (2006). Making therapeutic decisions in adults with aplastic anemia.. Hematology Am Soc Hematol Educ Program.

[8] Guinan EC (2009). Acquired aplastic anemia in childhood.. Hematol Oncol Clin North Am.

[9] Marsh JCW, Ball SE, Cavenagh J, Darbyshire P, Dokal I (2009). Guidelines for the diagnosis and management of aplastic anaemia.. Br J Haematol.

[10] Moher D, Liberati A, Tetzlaff J, Altman DG (2009). Preferred reporting items for systematic reviews and meta-analyses: the PRISMA statement.. PLoS Med.

[11] Liberati A, Altman DG, Tetzlaff J, Mulrow C, Gotzsche PC (2009). The PRISMA statement for reporting systematic reviews and meta-analyses of studies that evaluate health care interventions: explanation and elaboration.. PLoS Med.

[12] Cochrane (2005). Glossary of Terms (Version 4.2.5).

[13] MeSH (2006). MeSH Tree Number Changes - 2007 MeSH. September 14, 2006. Bone Marrorw Transplantation, deleted MN: E4.936.225.687.155.

[14] Tybaert S (2007). MeSH Data Changes - 2008.. NLM Tech Bull.

[15] Armand P, Antin JH (2007). Allogeneic stem cell transplantation for aplastic anemia.. Biol Blood Marrow Transplant.

[16] Bacigalupo A (2008). Treatment strategies for patients with severe aplastic anemia.. Bone Marrow Transplant.

[17] Davies JK, Guinan EC (2007). An update on the management of severe idiopathic aplastic anaemia in children.. Br J Haematol.

[18] ASH (2010). Bloodjournal of the American Society of Hematology, ASH Annual Meeting Abstracts: search terms = aplastic + anemia + transplantation.

[19] ClinicalTrials (2010). Clinical Trials: search terms = aplastic + anemia + transplantation.

[20] Schrezenmeier H, Bacigalupo A, Aglietta M, Frickhofen N, Führer M, Schrezenmeier H, Bacigalupo A (2000). Guidelines for Treatment of Aplastic Anemia. Consensus Document of a group of interntional experts.. Aplastic Anemia Pathophysiology and Treatment.

[21] Higgins JPT, Green S, Cochrane (2009). Section 13.5. Assessing risk of bias in non-randomized studies.. Cochrane Handbook for Systematic Reviews of Interventions Version 502 [updated September 2009].

[22] Parmar MK, Torri V, Stewart L (1998). Extracting summary statistics to perform meta-analyses of the published literature for survival endpoints.. Stat Med.

[23] Tierney JF, Stewart LA, Ghersi D, Burdett S, Sydes MR (2007). Practical methods for incorporating summary time-to-event data into meta-analysis.. Trials.

[24] Higgins JPT, Green S, Cochrane (2009). Section 9.4.3 A generic inverse-variance approach to meta-analysis.. Cochrane Handbook for Systematic Reviews of Interventions Version 502 [updated September 2009].

[25] Higgins JPT, Green S, Cochrane (2009). Section 7.7.7 Data extraction for estimates of effects.. Cochrane Handbook for Systematic Reviews of Interventions Version 502 [updated September 2009].

[26] DerSimonian R, Laird N (1986). Meta-analysis in clinical trials.. Control Clin Trials.

[27] Higgins JPT, Thompson SG, Deeks JJ, Altman DG (2003). Measuring inconsistency in meta-analyses.. BMJ.

[28] Higgins JPT, Green S, Cochrane (2009). Section 13.6.2.4 When pooling is judged not to be appropriate.. Cochrane Handbook for Systematic Reviews of Interventions Version 502 [updated September 2009].

[29] Higgins JPT, Green S, Cochrane (2009). Section 9.5.2 Identifying and measuring heterogeneity.. Cochrane Handbook for Systematic Reviews of Interventions Version 502 [updated September 2009].

[30] Thompson SG, Higgins JP (2002). How should meta-regression analyses be undertaken and interpreted?. Stat Med.

[31] Ahn MJ, Choi JH, Lee YY, Choi IY, Kim IS (2003). Outcome of adult severe or very severe aplastic anemia treated with immunosuppressive therapy compared with bone marrow transplantation: multicenter trial.. Int J Hematol.

[32] Arranz R, Otero MJ, Ramos R, Steegmann JL, Lamana ML (1994). Clinical results in 50 multiply transfused patients with severe aplastic anemia treated with bone marrow transplantation or immunosuppressive therapy.. Bone Marrow Transplant.

[33] Bacigalupo A, Hows J, Gluckman E, Nissen C, Marsh J (1988). Bone marrow transplantation (BMT) versus immunosuppression for the treatment of severe aplastic anaemia (SAA): a report of the EBMT SAA working party.. Br J Haematol.

[34] Bacigalupo A, Brand R, Oneto R, Bruno B, Socie G (2000). Treatment of acquired severe aplastic anemia: bone marrow transplantation compared with immunosuppressive therapy–The European Group for Blood and Marrow Transplantation experience.. Semin Hematol.

[35] Bayever E, Champlin R, Ho W, Lenarsky C, Storch S (1984). Comparison between bone marrow transplantation and antithymocyte globulin in treatment of young patients with severe aplastic anemia.. J Pediatr.

[36] Champlin R, Ho W, Bayever E, Winston DJ, Lenarsky C (1984). Treatment of aplastic anemia: results with bone marrow transplantation, antithymocyte globulin, and a monoclonal anti-T cell antibody.. Prog Clin Biol Res.

[37] De Planque MM, Richel DJ, Fibbe WE, den Ottolander GJ, Guiot HF (1990). Acquired severe aplastic anaemia in adults–a single centre study with 13 years follow-up.. Neth J Med.

[38] Doney K, Leisenring W, Storb R, Appelbaum FR (1997). Primary treatment of acquired aplastic anemia: outcomes with bone marrow transplantation and immunosuppressive therapy. Seattle Bone Marrow Transplant Team.. Ann Intern Med.

[39] Fouladi M, Herman R, Rolland-Grinton M, Jones-Wallace D, Blanchette V (2000). Improved survival in severe acquired aplastic anemia of childhood.. Bone Marrow Transplant.

[40] Führer M, Burdach S, Ebell W, Gadner H, Haas R (1998). Relapse and clonal disease in children with aplastic anemia (AA) after immunosuppressive therapy (IST): the SAA 94 experience. German/Austrian Pediatric Aplastic Anemia Working Group.. Klin Padiatr.

[41] Führer M, Rampf U, Baumann I, Faldum A, Niemeyer C (2005). Immunosuppressive therapy for aplastic anemia in children: a more severe disease predicts better survival.. Blood.

[42] Ghavamzadeh A, Iravani M, Vafaiezadeh F, Jahani M, Mousavi A (2004). Bone marrow transplantation versus immunosuppressive therapy in severe aplastic anemia, 1990–2001.. Arch Iran Med.

[43] Gillio AP, Boulad F, Small TN, Kernan NA, Reyes B (1997). Comparison of long-term outcome of children with severe aplastic anemia treated with immunosuppression versus bone marrow transplantation.. Biol Blood Marrow Transplant.

[44] Gluckman E, Devergie A, Faille A, Bussel A, Benbunan M (1979). Antilymphocyte globulin treatment in severe aplastic anemia–comparison with bone marrow transplantation. Report of 60 cases.. Haematol Blood Transfus.

[45] Halperin DS, Grisaru D, Freedman MH, Saunders EF (1989). Severe acquired aplastic anemia in children: 11-year experience with bone marrow transplantation and immunosuppressive therapy.. Am J Pediatr Hematol Oncol.

[46] Kahn Q, Ellis RJ, Skikne BS, Mayo MS, Allgood JW (2002). A retrospective analysis of long-term survival in severe aplastic anemia patients treated with allogeneic bone marrow transplantation or immunosuppressive therapy with antithymocyte globulin and cyclosporin A at a single institution.. Military Medicine.

[47] Kim I, Yoon SS, Park S, Kim BK, Kim NK (2003). The treatment of severe aplastic anemia: outcomes of bone marrow transplantation and immunosuppressive therapy in a single institution of Korea.. J Korean Med Sci.

[48] Kojima S, Horibe K, Inaba J, Yoshimi A, Takahashi Y (2000). Long-term outcome of acquired aplastic anaemia in children: comparison between immunosuppressive therapy and bone marrow transplantation.. Br J Haematol.

[49] Lawlor ER, Anderson RA, Davis JH, Fryer CJ, Pritchard SL (1997). Immunosuppressive therapy: a potential alternative to bone marrow transplantation as initial therapy for acquired severe aplastic anemia in childhood?. J Pediatr Hematol Oncol.

[50] Locasciulli A, van't Veer L, Bacigalupo A, Hows J, Van Lint MT (1990). Treatment with marrow transplantation or immunosuppression of childhood acquired severe aplastic anemia: a report from the EBMT SAA Working Party.. Bone Marrow Transplant.

[51] Locasciulli A, Oneto R, Bacigalupo A, Socie G, Korthof E (2007). Outcome of patients with acquired aplastic anemia given first line bone marrow transplantation or immunosuppressive treatment in the last decade: a report from the European Group for Blood and Marrow Transplantation (EBMT).. Haematologica.

[52] Paquette RL, Tebyani N, Frane M, Ireland P, Ho WG (1995). Long-term outcome of aplastic anemia in adults treated with antithymocyte globulin: comparison with bone marrow transplantation.. Blood.

[53] Pitcher LA, Hann IM, Evans JP, Veys P, Chessells JM (1999). Improved prognosis for acquired aplastic anaemia.. Arch Dis Child.

[54] Tzeng CH, Chen PM, Chuang MW, Liu JH, Hsieh RK (1989). Treatment of severe aplastic anemia: comparison of bone marrow transplantation to immunotherapy.. Chung Hua I Hsueh Tsa Chih.

[55] Viollier R, Passweg J, Gregor M, Favre G, Kühne T (2005). Quality-adjusted survival analysis shows differences in outcome after immunosuppression or bone marrow transplantation in aplastic anemia.. Ann Hematol.

[56] Werner EJ, Stout RD, Valdez LP, Harris RE (1989). Immunosuppressive therapy versus bone marrow transplantation for children with aplastic anemia.. Pediatrics.

[57] Gray R, Wheatley K (1991). How to avoid bias when comparing bone marrow transplantation with chemotherapy.. Bone Marrow Transplant.

[58] Wheatley K, Gray R (2004). Commentary: Mendelian randomization–an update on its use to evaluate allogeneic stem cell transplantation in leukaemia.. Int J Epidemiol.

[59] Ljungman P, Bregni M, Brune M, Cornelissen J, Witte TD (2009). Allogeneic and autologous transplantation for haematological diseases, solid tumours and immune disorders: current practice in Europe 2009.. Bone Marrow Transplant.

[60] Gafter-Gvili A, Ram R, Gurion R, Paul M, Yeshurun M (2008). ATG plus cyclosporine reduces all-cause mortality in patients with severe aplastic anemia–systematic review and meta-analysis.. Acta Haematol.

[61] Linden T, Gerss J, Jürgens H (2007). The crux of the log rank test.. Haematologica.

[62] Chan AW, Hrobjartsson A, Haahr MT, Gotzsche PC, Altman DG (2004). Empirical evidence for selective reporting of outcomes in randomized trials: comparison of protocols to published articles.. JAMA.

[63] Kirkham JJ, Dwan KM, Altman DG, Gamble C, Dodd S (2010). The impact of outcome reporting bias in randomised controlled trials on a cohort of systematic reviews.. BMJ.

[64] Song F, Eastwood AJ, Gilbody S, Duley L, Sutton AJ (2000). Publication and related biases.. Health Technol Assess.

[65] Egger M, Zellweger-Zahner T, Schneider M, Junker C, Lengeler C (1997). Language bias in randomised controlled trials published in English and German.. Lancet.

[66] Jüni P, Holenstein F, Sterne J, Bartlett C, Egger M (2002). Direction and impact of language bias in meta-analyses of controlled trials: empirical study.. Int J Epidemiol.

[67] Moher D, Pham B, Lawson ML, Klassen TP (2003). The inclusion of reports of randomised trials published in languages other than English in systematic reviews.. Health Technol Assess.

[68] Galandi D, Schwarzer G, Antes G (2006). The demise of the randomised controlled trial: bibliometric study of the German-language health care literature, 1948 to 2004.. BMC Med Res Methodol.

[69] Webb DK, Hann IM, Chessells JM (1991). Acquired aplastic anaemia: still a serious disease.. Arch Dis Child.

[70] Nissen C, Tichelli A, Gratwohl A, Warthmann C, Moser Y (1999). High incidence of transiently appearing complement-sensitive bone marrow precursor cells in patients with severe aplastic anemia–A possible role of high endogenous IL-2 in their suppression.. Acta Haematol.

[71] Speck R, Tichelli A, Gratwohl A, Nissen C (1994). Treatment of severe aplastic anemia: A longterm followup of 175 patients on antilymphocyte globulin or bone marrow transplantation.. Chin Med J.

[72] Tichelli A, Gratwohl A, Wursch A, Nissen C, Speck B (1988). Late haematological complications in severe aplastic anaemia.. Br J Haematol.

[73] Speck B, Gratwohl A, Nissen C, Osterwalder B, Wursch A (1986). Treatment of severe aplastic anemia.. Exp Hematol.

[74] Speck B, Gratwohl A, Nissen C, Osterwalder B, Signer E (1984). Treatment of severe aplastic anemia: a prospective study of antilymphocyte globulin versus bone marrow transplantation.. Prog Clin Biol Res.

[75] Speck B, Gratwohl A, Nissen C, Osterwalder B, Signer E (1983). Bone marrow graft versus ALG in patients with aplastic anaemia.. Biomed Pharmacother.

[76] Speck B, Gratwohl A, Nissen C, Leibundgut U, Ruggero D (1981). Treatment of severe aplastic anaemia with antilymphocyte globulin or bone-marrow transplantation.. Br Med J.

[77] Speck B, Gratwohl A, Nissen C (1980). Severe aplastic anemia: A prospective study on the value of different therapeutic approaches in 37 successive patients. Proceedings of the Annual Meeting of the European Foundation for Bone Marrow Transplantation, Sils Maria (Engadine), Switzerland, 13–16 April 1980.. Blut.

[78] Speck B, Gluckman E, Haak HL, van Rood JJ (1977). Treatment of aplastic anaemia by antilymphocyte globulin with and without allogeneic bone-marrow infusions.. Lancet.

